# A reservoir network model for sensory-guided probabilistic decision making

**DOI:** 10.1186/1471-2202-16-S1-P74

**Published:** 2015-12-18

**Authors:** Tomoki Kurikawa, Takashi Handa, Tomoki Fukai

**Affiliations:** 1 Brain Science Institute, RIKEN, Wako, Saitama, 351-0198, Japan; 2 Research Center Caesar, Bonn, 53175, Germany

## 

**Figure 1:**
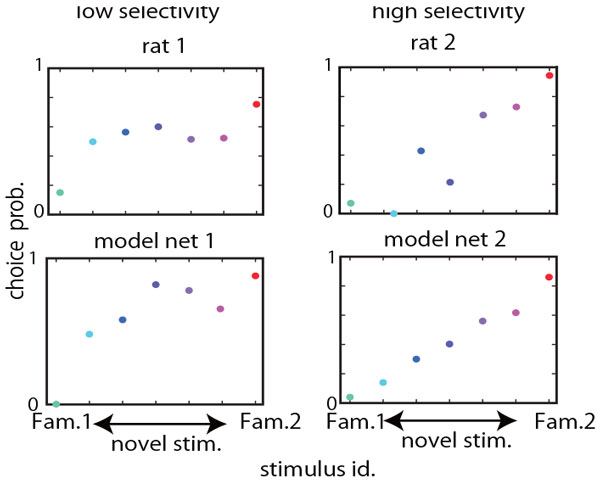


Animals are often required to adequately respond to novel stimuli on the basis of their previous sensory experiences. To investigate the neural mechanism underlying this sensory-guided decision making, we conducted multiunit recordings from the rats performing a two-alternative choice task. The rats were trained to make a LEFT or a RIGHT choice in response to two auditory cues, and then their behavior was tested for these familiar cues and other novel cues. Their choice probabilities generally varied in a graded manner such that the probability of choosing an option changed gradually according to frequency differences between familiar and novel cues. However, we also observed large variability in choice behavior across rats: choice probabilities for novel cues were near the chance level in some rats, while it systematically changed with tone frequency in other rats. We elucidated the mechanism underlying such decision behavior and the possible origin of individual behavioral differences in a neural network model (Fig. 1A). Our model is viewed as a reservoir network [1] and learns to associate two familiar cues with two alternative choices through reinforcement learning with eligibility trace [2].

Our model successfully replicated the gradual choice behavior observed in our experiment. We further analyzed how neural dynamics in the reservoir network determines the choice probability for familiar and novel cues. We found that a familiar stimulus sequentially activates a relatively small portion of reservoir neurons, and reinforcement learning trained output connections from these neurons such that only an adequate output neuron is activated by the neural trajectory evoked in the reservoir. We further revealed that choice responses to novel cues become graded due to trial-by-trial overlaps between the familiar trajectories and novel-cue-evoked trajectories.

Interestingly, our model also exhibited similar variability in choice responses to that observed in individual rats. We found that if input neurons are highly sensitive to external stimuli, that is, if the width of their frequency tuning curves is broad, the model network likely shows gradual choice behavior for novel stimuli. In contrast, the model with more sensitive input neurons (with broader tuning curves) tends to generate near-random choice behavior, displaying a flat dependence of choice probability on novel stimuli. We compared choice behavior between the models and the rats by introducing quantitative measures and found that the behavioral tendency of our model is consistent with that of the rats (Fig. 1B).

These results may suggest that some individual differences in decision making behavior emerge from neural population dynamics rather than differences in higher-level behavioral strategies.
